# Double-Staggered Grating Waveguide Slow Wave Structure for Terahertz Traveling Wave Tube

**DOI:** 10.3390/mi17020195

**Published:** 2026-01-31

**Authors:** Muhammad Haris Jamil, Nazish Saleem Abbas, Hamid Sharif, Wenlong He

**Affiliations:** 1 College of Electronics and Information Engineering, Shenzhen University, Shenzhen 518060, China; hamidnumls@gmail.com; 2 College of Physics and Optoelectronics, Shenzhen University, Shenzhen 518060, China; nazsa.786@gmail.com

**Keywords:** double staggered grating, slow wave structure, sheet electron beam, traveling wave tube

## Abstract

A double-staggered grating waveguide slow wave structure (DSGW–SWS) is designed for a 340 GHz traveling wave tube (TWT). Input and output couplers were also designed to isolate the electron beam source from the electromagnetic (EM) signal. Transition sections in the SWS circuits were made by tapering the height of the DSWG to improve the matching of the circuit with the couplers. The reflection coefficient has a wide range from 326 GHz to 364 GHz below −15 dB. Particle-in-cell (PIC) simulation is performed using an ideal particle source for sheet electron beam (SEB), considering the filling factor to be around 50%. The average input power of a 340 GHz signal is said to be 0.19 W, which is amplified to 17.4 W with a gain of 19.55 dB.

## 1. Introduction

Vacuum electronic devices (VEDs) constitute the most remarkable high-power oscillators and amplifiers at the Terahertz frequency domain [1,2,3,4]. One of the significant VEDs is a traveling wave tube (TWT), which holds a broad range of applications in commercial and military sectors because of its large bandwidth and high power at high frequencies. An important part of a traveling wave tube is the slow wave structure (SWS) for beam–wave interaction. Optimizing the SWS to increase the interaction impedance and reduce the phase velocity of the wave results in better beam–wave interaction, hence increasing the output power of the TWT. Commonly used slow wave structures include double-staggered grating waveguides [5,6,7], sine waveguides [8,9] and folded waveguides [10,11,12].

In terms of operational bandwidth, the double-staggered grating waveguide (DSGW) has an advantage over the folded waveguides and sine waveguides. In a previous study [13], an optimized 220 GHz double-staggered grating waveguide (DSGW) amplifier produced 67 W of output power with a 3 dB bandwidth of 31.5 GHz, operating at a 25 kV beam voltage and 80 mA beam current. A 200 GHz DSGW operating at 20 kV and 100 mA was reported in Ref. [14]. A peak gain achieved with a 40 mm long tube was 18.9 dB. In Ref. [10] a staggered double-segment grating SWS achieves 32.8 W at 340 GHz with a 19.2 kV, 60 mA beam. Nevertheless, its intricate three-dimensional geometry is difficult to fabricate, especially when contrasted with the simpler planar alternative presented here in the article. In Ref. [15], a 0.34 THz traveling wave tube is discussed with an output power of 25 W and a gain of 40 dB but having a shorter bandwidth of 10 GHz. A folded waveguide TWT having an output power of 3.1 W at 336.96 GHz and a corresponding gain of 26.2 dB is reported in Ref. [16]. The amplification is achieved by the interaction of a cylindrical electron beam of 16.2 kV and 25.2 mA with an RF signal of 7.2 mW.

In this article we will discuss the design of a staggered double grating slow wave structure and H-plane 90-degree bend input–output couplers with Bragg reflectors for a 340 GHz traveling wave tube amplifier. The description of the SDG-SWS unit cell and its electromagnetic properties are discussed in Section 2. In Section 3 the transmission characteristics of the SWS and the coupler are discussed. The beam–wave interaction is studied using the computer simulation technology particle-in-cell (CST-PIC) solver, and the results are presented in Section 4. The conclusion and some results are discussed in Section 5.

## 2. Unit Cell and Electromagnetic Properties

**Figure 1:**
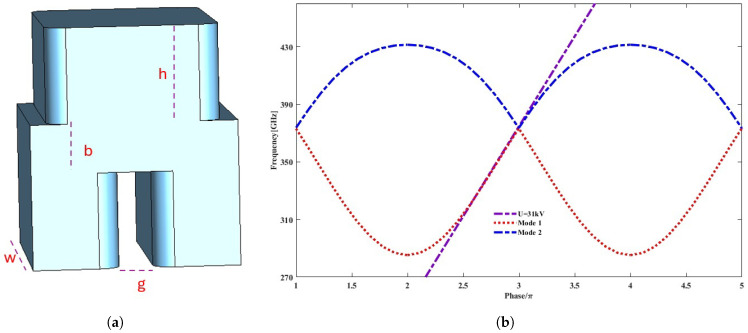
(**a**) Unit cell of SDGW. (**b**) Dispersion curve.

The unit cell of a staggered double grating slow wave structure is shown in Figure 1a. The optimized parameters of the unit cell are given in Table 1. The CST studio eigenmode solver is used to optimize the unit cell parameters and to achieve a linear dispersion curve. Figure 1b shows the dispersion curve of the unit cell with two fundamental modes. To obtain the operating frequency of each mode, the axial phase shift in each cell is swept from π to 5π in eigenmode simulation. It can be seen that the beam voltage line of 31 kV matches with dispersion curves of both mode 1 (forward wave) and mode 2 (backward wave) for a wide frequency range of 319 GHz to 373 GHz and 373 GHz to 397 GHz, respectively, making it eligible for dual-mode operation.

Another important figure of merit in the design of a TWT–SWS is the interaction impedance expressed as

(1) $$k_{c}=\frac{\left|E_{z,n}\right|}{2P\beta_{n}^{2}}$$

**Figure 2:**
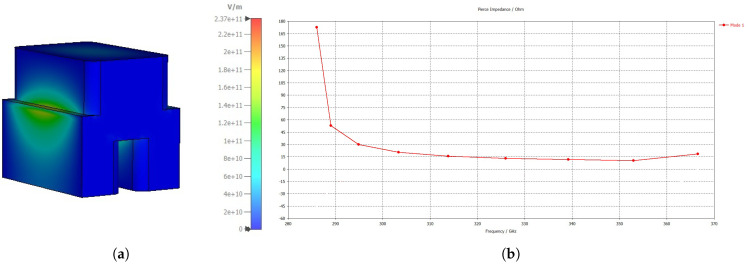
(**a**) Longitudinal electric field of Mode 1. (**b**) Interaction impedance.

It gauges the coupling strength between the waveguide mode and the electron beam. The interaction impedance is a quantifier of how effectively an electron beam interacts with the axial component of the propagating electromagnetic wave. Therefore, the higher the interaction impedance, the higher the energy transfer from the electron beam. Figure 2 shows the longitudinal electric field intensity and the interaction impedance of the TM11-like mode.

## 3. Transmission Analysis of SWS

The slow wave structure comprises 100 optimized unit cells repeated along the axial direction (z-axis). In the DSG slow wave structures, the electromagnetic power cannot be decoupled from the beam under normal interaction conditions. Hence, designing an efficient coupling structure is critical. The power can be taken out of the tube either in a vertical or horizontal direction with respect to the beam plane. Therefore, 90-degree bend H-plan input–output couplers are used for this purpose. Bragg reflectors at the ends of the couplers are there to avoid the loss of RF signal into the electron gun.

**Figure 3:**
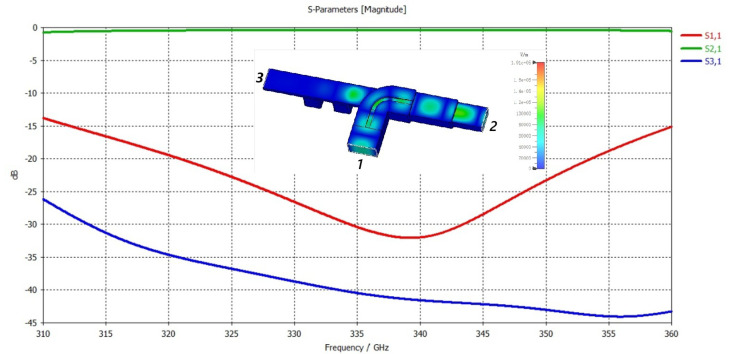
Electric field and s-parameters of coupler.

Ohmic losses from surface roughness are modeled via the Hammerstad–Bekkadal formula expressed in Equation (1) to accurately predict frequency-dependent bulk conductivity under realistic conditions.

(2) $$\begin{array}{cc} \sigma = & \sigma_{0}{\left\{1+\frac{2}{\pi }\arctan\left[1.4{\left(\frac{h}{\delta }\right)}^{2}\right]\right\}}^{-2} \end{array}$$

where $\sigma_{0}$ is vacuum conductivity, *h* is the surface roughness of the material and δ is the skin depth. If the roughness of the copper is 300 nm at 340 GHZ, the conductivity is calculated to be 1.53 × 10^2^ S/m. Considering these values, the time-domain solver simulation of the coupler is performed to find the s-parameters. Figure 3 shows that the reflection loss $S_{1,1}$ is less than −15 dB and the $S_{2,1}$ is greater than −1 dB for a frequency ranging from 311 GHz to 360 GHz, making the coupler well eligible to be used in the circuit.

**Figure 4:**
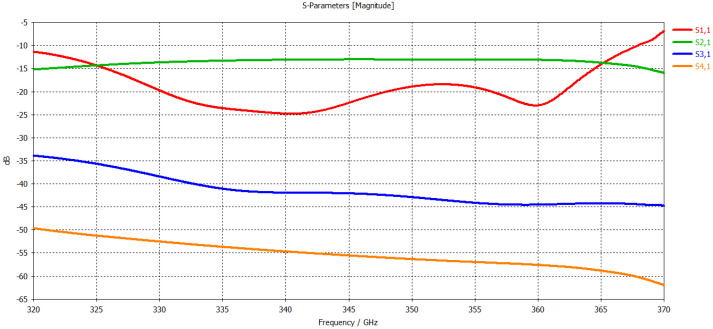
S-parameters of the SWS circuit along with the input and output coupler.

The three unit cells at both the input and output sides of the circuit are tapered to make a transition section, which optimizes SWS-to-coupler impedance matching at both ends. Hence, improving the transmission characteristics of the SWS by reducing mismatch losses. The total length of the SWS, including the transition section, is 40 mm. Results of the time-domain solver simulation considering the above-mentioned roughness and conductivity are shown in Figure 4. The design achieved a good impedance matching with $S_{1,1}$ below −15 dB from 326 GHz to 364 GHz, making an operational bandwidth of 38 GHz.

## 4. Beam–Wave Interaction

**Figure 5:**
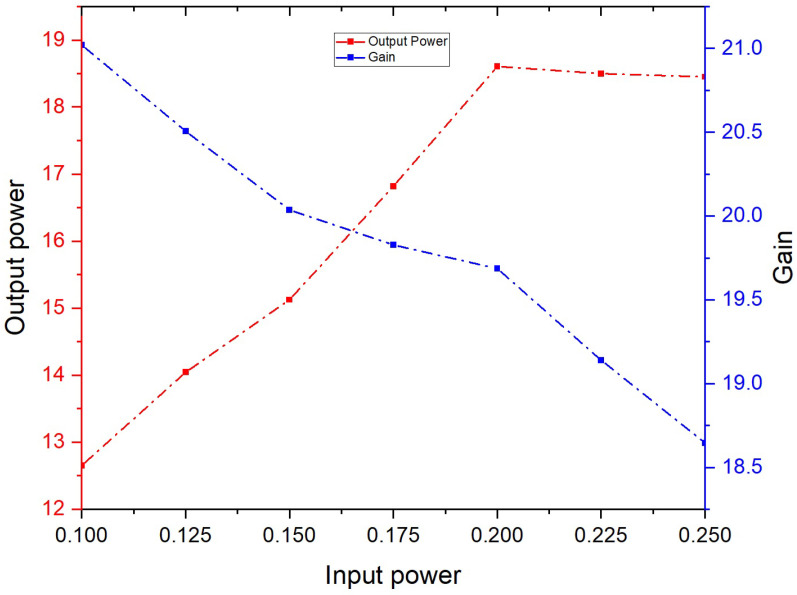
Input power vs. output power and gain.

The CST particle studio is used for PIC simulation for evaluating the hottest characteristics of the above-discussed SWS in a TWT. This simulation considers the overall circuit consisting of the SWS and the input–output couplers. The cross-section size of the particle source used is 0.48 × 0.052 mm^2^. The beam used has a current of 40 mA and a voltage of 31 kV. An analytic magnetic field with an axial component of 0.6 T is used to confine the beam. The small signal gain of the circuit is kept near 20 dB to avoid oscillations. Particle-in-cell simulation is performed for an input signal of frequency 340 GHz with different input power levels. The length of the circuit is kept constant with 100 periods of SWS unit cells as considered for the transmission analysis in the above section. In Figure 5, a graph between input power, output power and gain is shown. A saturated average power of 18.6 W and a gain of 19.6 dB is achieved for the input signal of 0.21 W, as depicted from the graph.

**Figure 6:**
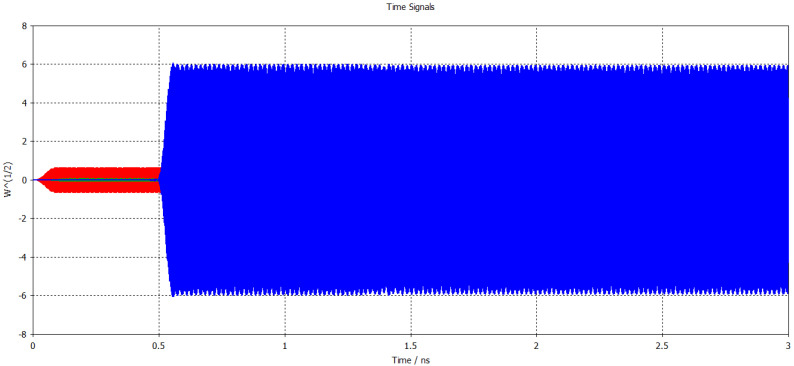
Input–output signal versus time at 340 GHz.

Considering the saturation condition from the above graph and to avoid the oscillations, a time domain simulation with the average input power of 0.19 W was performed. The result of this simulation is shown in Figure 6, where the output amplified signal with a peak power of 5.8 W can be seen. It is depicted that the output signal becomes stable at 0.55 ns, generating an average output power of 17.4 W. Based on this result, the gain of the circuit is calculated to be 19.55 dB.

**Figure 7:**
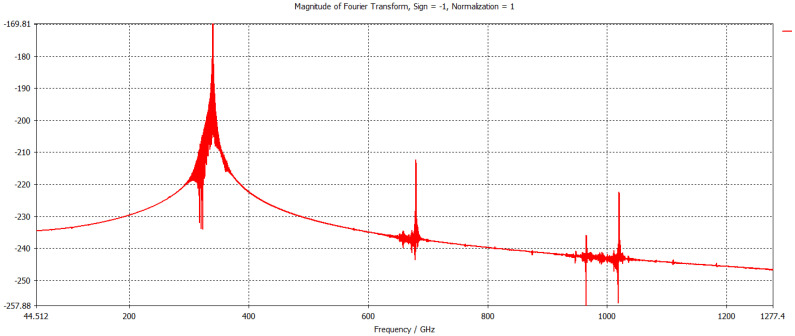
Fourier spectrum of the output signal.

The frequency spectrum of the output signal is illustrated in Figure 7, showing a stable spectrum concentrated at frequency 340 GHz. The normalized amplitude at the other frequency points is quite minimal, which shows that the competing modes are well suppressed.

**Figure 8:**
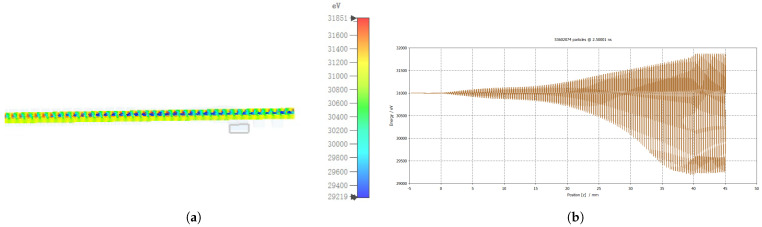
(**a**) Bunching effect; (**b**) energy vs. phase graph.

Longitudinal bunching within the tube, shown in Figure 8a, arises from velocity modulation of the electron beam due to its interaction with the input signal’s electromagnetic field. The accompanying longitudinal energy distribution provides additional evidence of this process. Figure 8b clearly demonstrates electron deceleration toward the tube’s end, indicating energy transfer from the beam to the EM wave and resulting in signal amplification.

## 5. Conclusions

The design of a double-staggered grating waveguide for a 340 GHz frequency signal is discussed in this paper. The SWS has been designed by optimizing the unit cell to achieve better interaction impedance. Input and output couplers are also designed as a part of the circuit to isolate the electron beam from the high-frequency circuit. The transmission characteristics show a good passband of 38 GHz for the DSGW. The hottest characteristics of the tube are also studied to analyze the beam–wave interaction in detail.

## Figures and Tables

**Table 1:** Dimensional values of SWS unit cells.

*h*	*b*	*w*	*g*
0.185 mm	0.095 mm	0.53 mm	0.065 mm

## Data Availability

The data that support the findings of this study are available from the corresponding author upon reasonable request.
